# Study of Saiga Horn Using High-Performance Liquid Chromatography with Mass Spectrometry

**DOI:** 10.1100/2012/759604

**Published:** 2012-05-02

**Authors:** Kateřina Mikulíková, Oleg Romanov, Ivan Miksik, Adam Eckhardt, Statis Pataridis, Pavla Sedláková

**Affiliations:** ^1^Institute of Physiology, Academy of Science of the Czech Republic, VÍdeňská 1083, Prague, Czech Republic; ^2^Kalmykian State University, Elista, Russia; ^3^Cardiovascular Research Centre, Prague, Czech Republic

## Abstract

The saiga horns have been investigated the using of modern analytic methods. High-performance liquid chromatography (HPLC) with mass-spectrometric (MS and MS/MS) detection and polyacrylamide gel electrophoresis (PAGE) were used. It could be concluded that basic proteins of the saiga horns are keratins and collagen. The basic representation protein in all samples is keratin type I microfibrillar (from sheep), keratin type II microfibrillar (from sheep), collagen type I (*α*_1_) (from bovine) and collagen type I (*α*_2_) (from bovine). Free amino acids we determined in all samples are nontreated by enzyme.

## 1. Introduction

This work is devoted to the idea of research of alternative to the active components in the horns of Saiga. Composition of horns of Saiga is studied for this purpose, in future to find the analogues of active components in the horns of other animals, for example, ram. Saiga antelope (*Saiga tatarica tatarica*) populations are concentrated in three main areas within central Asia: Mongolia, Kazakhstan, and Kalmykia (Russia RF). *Saiga tatarica tatarica* inhabits dry steppes and semideserts. Herds are found in grassy plains void of rugged terrain and hills. The most striking feature of a saiga is its large head with a huge mobile nose that hangs over its mouth. Males have a pair of long, waxy-colored horns with ring-like ridges along their length. Except for the unusual snout and horns,* S*. *tatarica tatarica *looks similar to small sheep. Saiga antelopes are approximately 0.6 to 0.8 m tall at shoulder height and are approximately 1 to 1.5 m long [1]. They have long, thin legs, a slightly robust body, and a short tail. In the early 1990s in an effort to reduce the losses of rhinoceros to poaching, conservation organizations advocated the use of saiga antelope horn in Traditional Chinese Medicine. By 2001 the saiga had joined the rhinoceros on the endangered species list. Many nonendangered alternatives were available but not promoted. The saiga, an animal that is actually somewhere between antelope and wild sheep genetically, has historically rebounded from population crashes caused by severe weather including droughts and harsh winters. The species has a very short lifespan −10 years at the most, so losing even one reproductive season causes serious population impacts.

Horn is a hard animal tissue related to hair, with a rigid structure due to the sulphur cross-linkages in the protein kreatin [2]. In the opinion of ancient doctors, head is the focus of all forces of organism, and their greatest concentration is in the horns. Horns indicate supernatural force, deity, force of spirit, or the vital principle, which appears from the head. Horns of saiga, rhinoceros, and buffalo were as damping, antipyretic, fever-lowering medicine. Fundamentals components of any horns is keratin, free amino acids, peptides, lipids, nuclein acids, remain microelements: calcium, aluminium, chromium, copper, iron, manganise, and zinc [3].

Keratin proteins comprise a major portion of the protective matrix of the skin, hair, horn, beak, and feathers of mammals and fowl [4]. Keratin proteins provide the structural basis for the unique properties of the biomaterial horn and its protective function against a wide range of environmental factors. When nutrient supplied to keratin-forming cells is compromised or completely interrupted, inferior keratinized tissue, that is, horn, is produced, which may lead to increased susceptibility to claw disorders and ultimately to lameness [5]. Keratins are a broad class of fibrous proteins [6]. The two major classes of keratins have been termed *α*-keratins and *β*-keratins. Although these keratins can be characterized based on genomic, chemicophysical, ultrastructural, and morphological characters [7, 8], an intrinsic difference in vertebrate keratins is that reptiles and birds produce both *α*- and *β*-keratins, whereas mammals produce only *α*-keratins [7–12]. While keratin nomenclature can be confusing [13–16], *α*-keratins are characterized by a helical structure, whereas *β*-keratins have a pleated structure. The primary role of keratin is to make the skin, hair, and horn a pliable, insoluble, and unreactive barrier against the natural environment. There are a lot of varied fibrous structural proteins of the epidermis collectively termed keratin proteins. Keratin is often misunderstood as a single substance even though it is composed of a complex mixture of proteins. The presence of the following substances in keratinizing cells serves as a positive indicator of intense cellular activity: ribonucleic and deoxyribonucleic acid, ascorbic acid, free aldehyde groups, alkaline phosphatase, lipids, glycogen, and glutathione [4, 17]. Keratins are normally differentiated as “soft” or “hard,” corresponding to the products of two apparently. Soft keratins occur in the stratum corneum, corns, calluses, and the eponychium (coronary band) around the hoof, whereas hard keratins are found in hair and horn. Either form of keratinization produces extremely hard keratin (turtle shell scutes, horn sheaths, hoof ungues, claw ungues, etc.), which provides external protection to mammals and reptiles [12, 18]. The amino acids Cys, His, and Met play key roles in establishing the structural integrity of the keratinocyte [19, 20]. Zinc has been identified as a key mineral in the processes of keratinization [5, 21]. Zinc also plays a key role in the formation of the structural proteins during the keratinization process. Zinc-finger proteins are involved in functions requiring protein-to-protein interactions, most of which are thought to affect cellular differentiation or proliferation [22]. Vitamins also play an integral role in developing the structure and quality of keratinized horn tissue (A, D, E, biotin) [23].

Modern analytical methods, especially combination HPLC with mass spectrometry, were used for the bioanalysis of peptides and proteins in biological samples.

Keratin in animal horn and hoof and in feathers is hard-fiber protein, and its treatment is very difficult. They are therefore presently only used as feed and foaming agents for fire extinguishers [24]. Kida et al. [25] developed an conditions for enzymatic hydrolysis to effectively utilize horn and hoof and were able to produce enzymatic hydrolysates from them. Ram horns, known as fibrous protein source are widely produced across the world. In Turkey, more than 600 tons of horn are produced per year. Furthermore, other fibrous proteins from feathers, nails, hair, and so forth are available as waste [26, 27]. These waste products can be converted to biomass, protein concentrate, or amino acids using proteases derived from certain microorganisms [28]. Ram horns consist of *α*-keratin, which is relatively rich in cysteine (up to 22%). Keratins have been studied with various forms of infrared spectroscopy for decades [29]. Edwards et al. [30] differentiated horn and turtle keratin with Raman spectroscopy though their sample sizes were relatively small. A broad range of sea turtle and bovid species, showing that diffuse reflectance infrared [31] compared results from two FT-IR methods, attenuated total reflectance (ATR) and DRIFTS, and found that DRIFTS provided better discrimination and quantitative results than the ATR method. Fourier transform spectroscopy (DRIFTS), combined with discriminant analysis, is a useful quantitative tool for distinguishing their keratins.

A chemical analysis of saiga horn has revealed that the main constituents are keratin, calcium phosphate, and vitamin A. As saiga horn has a high zinc content, it is believed that consumption of saiga horn would help to delay ageing. Amino acid composition of saiga horn has been already described [32]. 

## 2. Material and Methods

### 2.1. Chemicals

All chemicals used were either of analytical grade or the highest available purity. All solutions were prepared in MilliQWater (Millipore, Bedford, MA, USA); EDTA (ethylenedinitrilotetraacetic acid, disodium salt) from Merk, Darmstadt, Germany; guanidine hydrochloride from AppliChem, Darmstadt, Germany; BIS (*N*, *N*-methylenebis-acrylamide), and acrylamide was obtained from Sigma (St. Louis, MO, USA); ammonium persulfate (APS); bromophenol blue (3,3,5,5-tetrabromophenolsulfonephthalein); tetramethylethylenediamine (TEMED); 2-mercaptoethanol; glycerol, SDS and the low molecular mass protein markers (albumin, ovalbumin, glyceraldehyde-3-phosphate dehydrogenase, carbonic anhydrase, trypsinogen, trypsin inhibitor, *α*-lactalbumin, aprotinin), iodoacetic acids, DL-dithiotreitol were purchased from Sigma as well. Crystallized and lyophilized trypsin, tris (hydroxymethyl) aminomethane hydrochloride (tris-HCl), ammonium bicarbonate from Sigma; all other chemicals were purchased from Lachema (Brno, Czech Republic) in an analytical grade (p.a.).

### 2.2. Instruments

The HPLC apparatus used was an HP 1100 LC system (Agilent, Palo Alto, CA, USA) consisting of a degasser, a binary pump, an autosampler, a thermostated column compartment, and a diode array detector. The instrument was controlled, and the data collected and manipulated by the program ChemStation B.01.03. It was coupled to an ion-trap mass spectrometer (Agilent LC-MSD Trap XCT-Ultra); for details on the instrument conditions, see conditions for HPLC-MS. Analysis of MS/MS data (peptide/protein identification) was carried out using the software SpectrumMill (v.3.02, Agilent). The searches were performed in the full protein databases SwissProt and NCBI and then on the data extracted from these databases.

### 2.3. Sample Preparation

#### 2.3.1. Tissues and Animals Used

Three types of saiga horn samples were used (for details see Table 1):

**(a) Different Parts of Horn**

Lowest part versus 2/3 part of horn for 2.5 years old saiga (2.5 DGN versus DGV II^2^), and lowest part versus top part of horn for 3-year old saiga (3 DGN versus 3 DGV).

**(b) Rut (Age)**

before and after for 1.7-year old saiga versus top part of horn (1.7 PGV versus 1.7 DGV).

**(c) Age**

2.5 years versus 3-year old saiga for lowest part of horn (2.5 DGN versus 3 DGN) and 1.7 versus 3 years old for top part of horn (1.7 DGV versus 3 DGV).

#### 2.3.2. Sample Preparation

Saiga horns were homogenized by milling in a mill. Shavings were extracted by a 50% EtOH. Solution was evaporated to dryness under vacuum. Only sample-labeled DGV II^2^ was fractionated with Sephadex G-25 then purified on BioGel RP-2.

#### 2.3.3. Enzymatic Digestion

Samples (5 mg of 2.5 DGN; 1 mg of DGV II^2^; 1 mg 1.7 PGV; 1 mg 1.7 DGV; 1 mg 3 DGN; 1 mg 3 DGV) were dissolved in 0.5 mL of 6.0 M guanidine HCl, 1.2 M Tris/HCl, 2.5 mM Na_2_EDTA (pH 8.4) buffer than were reduced disulfides by adding 25 *μ*L of 1.0 M DTT and placing at 65°C for 30 min. S-Carboxymethylation (alkylation) was done by adding 60 *μ*L of 1.0 M iodoacetic acid and placing at room temperature, in the dark, for 40 min. Alkylation was stopped by adding 15 *μ*L of 1.0 M DTT (dithiotreitol) and desalted and exchange into digestion buffer by applying reaction mixture to Econo-Pac 10 DG columns equilibrated with 20 mM NH_4_HCO_3_ pH 7.8 digestion buffer. Carboxymethylated protein was eluted and collected by digestion buffer. Treated proteins were digested by adding trypsin (1 : 50 enzyme ratio: substrate) and incubated at 37°C for 3 h and analysed by HPLC/MS.

### 2.4. Separation Conditions

#### 2.4.1. Conditions for HPLC-MS/MS

Chromatographic separation was carried out on a Jupiter Proteo 90 A, 250 mm × 2 mm (Phenomenex, Torrance, CA, USA). Separation was achieved by a linear gradient between mobile phase A (H_2_O + 0.1% HCOOH) and B (CH_3_CN + 0.085% HCOOH). Separation was initiated by running the system isocratically for 2 min with 2% mobile phase B, followed by a gradient elution to 35% B at 40 min. Finally the column was eluted with 100% B for 50 min. Equilibration before the next run was achieved by washing with 2% of mobile phase B for 10 min. The flow rate was 0.25 mL/min, the column temperature was held at 25°C, and UV absorbance detection was done at 214 nm. Atmospheric pressure ionization-electrospray ionization- (API-ESI) positive mode ion-trap mass spectrometry was used. Separation was achieved by a linear gradient between mobile phase A (H_2_O) and B (CH_3_CN) for atmospheric pressure chemical ionization (APCI). Operating conditions, drying gas (N_2_), 10 l/min; drying gas temperature, 350°C; nebulizator pressure, 25 psi; ions were observed over the mass range m/z 100–2200 (MS-standard mode, MS/MS-enhanced mode). Analysis was done in auto-MS/MS mode (10 precursor ions, excluded after 2 spectra for 0.5 min). The spectrum-mill autovalidation of spectra was performed using default settings, but all spectra were then evaluated manually.

#### 2.4.2. Polyacrylamide Gel Electrophoresis

A further insight into the analyses of saiga horn samples is polyacrylamide gel electrophoresis. These samples were dialyzed before analysis. The separating gel (10%) was prepared by mixing 2.45 mL water, 1.25 mL Tris-HCl (1.5 mol/L, pH 8.8), 50 *μ*L SDS (10% (w/v)), 1.25 mL acrylamide (30% (w/v)), and bismethylenacrylamide (0.8% (w/v)), 25 *μ*L APS (10% (w/v)), and 2.5 *μ*L TEMED. This gel was polymerized for 45–60 min at room temperature. Next the stacking gel (4%) was prepared by mixing 3.05 mL water, 1.25 mL Tris-HCl (0.5 mol/L, pH 6.8), 50 *μ*L SDS (10% (w/v)), 0.65 mL acrylamide (30%) and bis-methylenacrylamide (0.8%), 25 *μ*L APS (10% (w/v)), and 5 *μ*L TEMED. This gel polymerized within 35–45 min. After the samples, and 5 *μ*L of protein standards (molecular weight range 6.5–205 kDa, Sigma), were applied, electrophoresis was run at 150 V over the gel. Finally the gel was stained by a solution of Coomassie brilliant blue (0.25% (w/v)) and destained in 1% (v/v) acetic acid for 120 min. The resulting separation was scanned by flat scanner (hp scanjet 7400c; Hewlett-Packard).

## 3. Results and Discussion

### 3.1. HPLC-MS/MS

**Figure 1:**
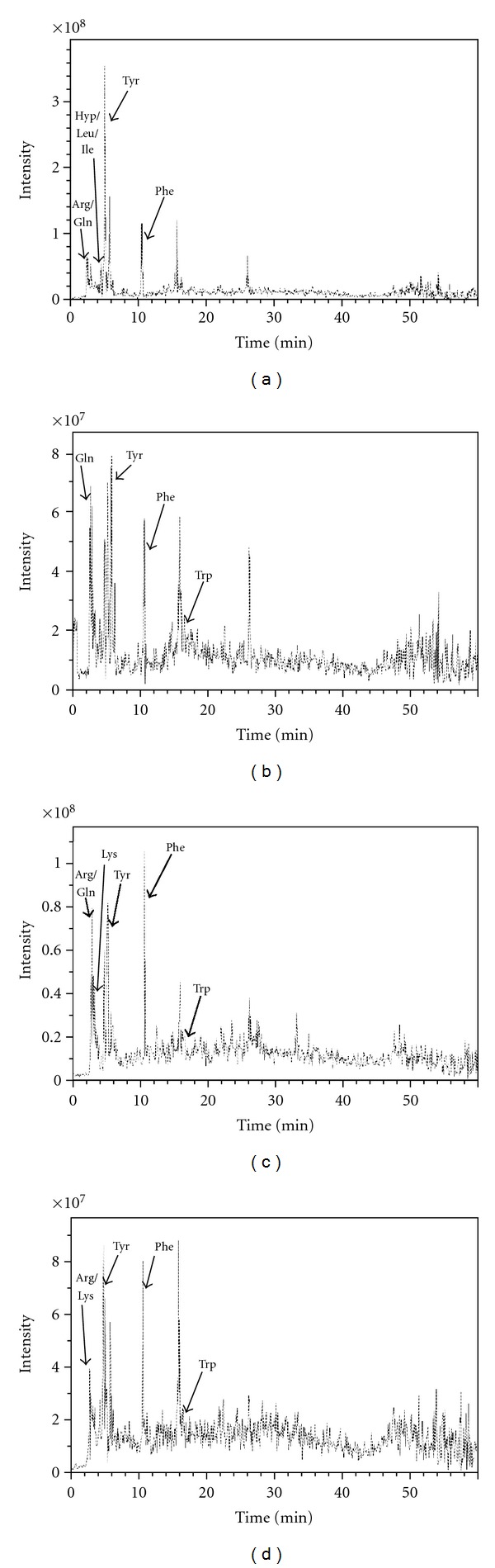
Separation of nontreated horn samples by HPLC/MS: (a) 1.7 PGV identification free amino acids: Arg/Gln (arginine/glutamine); Hyp/Leu/Ile (hydroxyproline/leucine/isoleucine); Tyr (tyrosine); Phe (phenylalanine), (b) 1.7 DGV glutamine; tyrosine; phenylalanine; Trp (tryptofan), (c) 3 DGV arginine/glutamine; Lys (lysine); tyrosine; phenylalanine; tryptofan, (d) 3 DGN arginine/lysine; tyrosine; phenylalanine; tryptofan.

**Figure 2:**
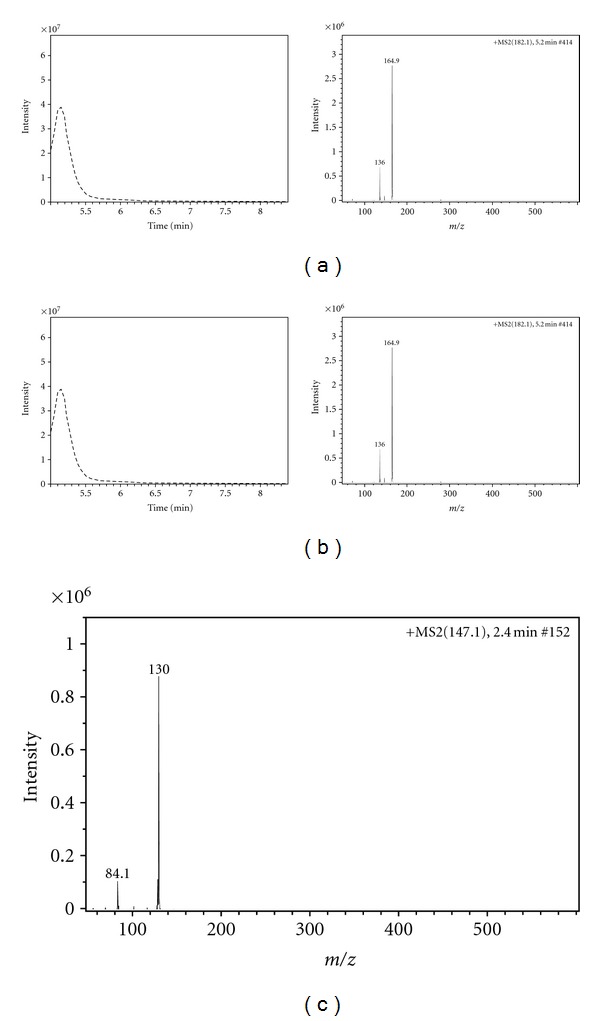
Mass spectrum of nontreated 1.7 PGV. (a) extraction ion current (EIC) of 120 + MS 2 (166) m/z for phenylalanine, (b) extraction ion current (EIC) of 165 + MS 2 (182) m/z for tyrosine, (c) extraction ion current (EIC) of 130 + MS 2 (147.1) m/z for glutamine.

Nontreated (i.e., without enzymatic cleavage) saiga horn samples were analysed by HPLC-coupled APCI-MS. There were detected free amino acids (arginine, glutamine, hydroxyproline, leucine, isoleucine, tyrosine, phenylalanine, tryptofan, and lysine). These results were confirmed by analysis of amino acid standards. Example of mass spectra (MS/MS) of individual amino acids in sample is demonstrated in Figure 1. MS and MS/MS analysis allows to identify individual amino acids. Chromatographic profiles of nontreated samples from saiga horn are presented in Figure 2. Amino acids tyrosine and phenylalanine were found in all samples, while hydroxyproline, leucine, and isoleucine were discovered only in sample 1.7 PGV. This content of hydroxyproline reflects the highest detectable amount of collagenous peptides in this sample (see next text). Only the sample 1.7 PGV contains amino acid tryptofan. The sample 1.7 DGV does not contain amino acid arginine. Amino acid lysine was found only in sample 3 DGN and 3 DGV. Only the sample 3 DGN does not contain amino acid glutamine.

**Figure 3:**
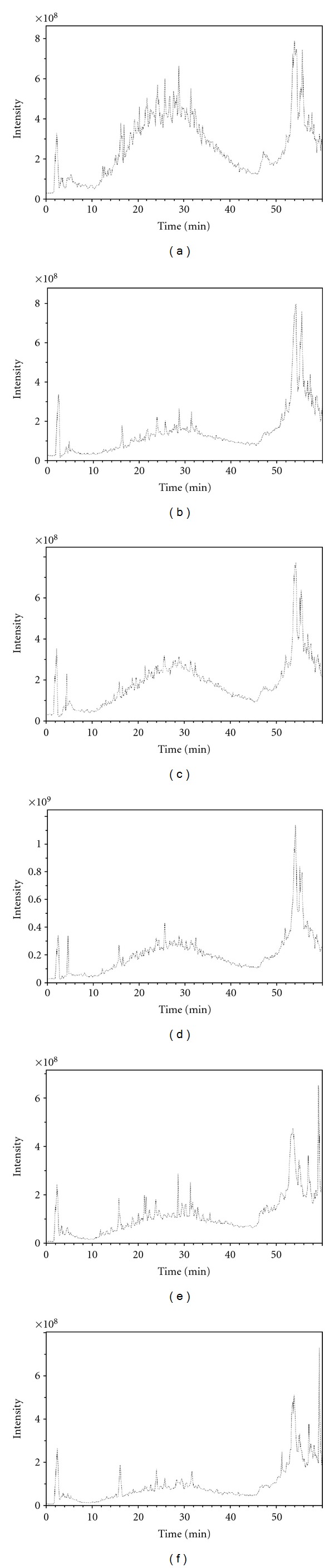
HPLC/MS analysis of horn samples digested by trypsin (a) 1.7 PGV, (b) 1.7 DGV, (c) 3 DGV, (d) 3 DGN, (e) 2.5 DGN, and (f) DGV II^2^.

**Figure 4:**
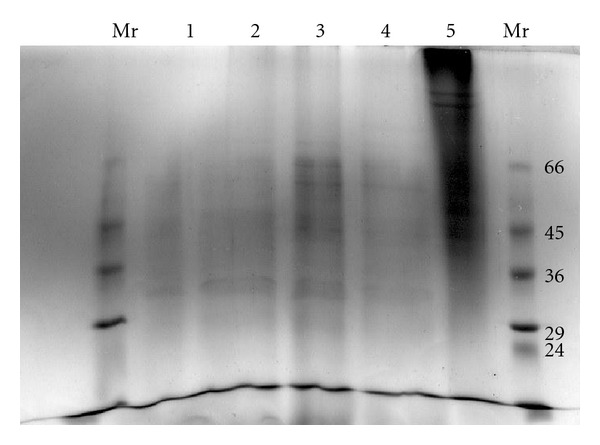
Polyacrylamide gel electrophoresis (PAGE) analysis of saiga horns protein. Left lane: low Mr markers: Albumin, bovine serum (Mr 66,000), Ovalbumin, chicken egg (Mr 45,000), Glyceraldehyde-3-phosphate dehydrogenase, rabbit muscle (Mr 36,000), Carbonic anhydrase, bovine erythrocytes (Mr 29,000), Trypsinogen, bovine pancreas (Mr 24,000), Trypsin Inhibitor, soybean (Mr 20,000), *α*-Lactalbumin (Mr 14,200), Aprotinin, bovine lung (Mr 6,500); (lane 1) 1.7 PGV; (lane 2) 1.7 DGV; (lane 3) 3 DGN; (lane 4) 3 DGV; (lane 5) collagen type I; and right lane: low Mr markers.

HPLC-MS/MS analysis of saiga horn samples treated by trypsin gave up peptide maps of proteins. HPLC-ESI-MS (MS/MS) profiles of tryptic peptides are shown in Figure 3. Polyacrylamide gel electrophoresis of saiga horn protein is shown in Figure 4. It is obvious broadening of the zone. This appears indicative of several incompletely resolved molecular species present in the zones corresponding to 1.7 PGV, 1.7 DGV, 3 DGV, and 3 DGN.

HPLC-MS/MS analysis allows to identify several proteins of saiga horn based on the identification of specific peptides (Table 2).

At the Table 2 it is obvious that all samples of saiga horn contain this basic protein: keratin type I microfibrillar 48  kDa, component 8C1 (from *Ovis aries*, sheep), keratin type II microfibrillar, component 5 (from *Ovis aries*, sheep) except the sample 3 DGV and DGV II^2^, collagen *α*_1_ (I) chain precursor (from *Bos taurus*, bovine), collagen *α*_2_ (I) chain precursor (from *Bos taurus*, bovine) except the sample 3 DGV and 2.5 DGN, and the sample 3 DGN does not contain collagen *α*_2_ (I). The sample 1.7 PGV contains the most proteins and peptides of saiga horn. In this sample it is also the most abundant protein of extracellular matrix (connective tissue)—collagen type I. It should be mentionied that proteins of saiga antelope are not commonly included in databases searched. For this research proteins detectable are devoted to sheep or other animals.

As it was mentioned above, saiga antelope horns were not analysed by proteomic research previously. There is only a limited knowledge above protein content of horns. Park et al. [33] obtained that overall image of antler proteome used 2-DE gel. From this 2-DE gel, ~300 spots were excised and subjected to in-gel trypsin digestion and subsequent PMF analysis by MALDI-TOF MS. They were able to identify about 95–100 protein spots. The identified antler proteins are diverse and can be grouped into several categories according to their functions. Among their discovered proteins that belong to structural proteins are keratin type I microfibrillar, collagen type *α*_1_ (II), keratin type II cytoskeletal 8, and collagen type *α*_1_ (I). Further they examined the difference in proteome profiles between growing and ossified parts of antlers. They identified several of proteins to be implicated in cell growth (collagen *α*_2_ (I) and zinc finger protein 28 homolog). Similarly we identified in the sample 1.7 PGV (1.7 years old saiga, after rut) zinc finger protein 643, keratin type I microfibrillar, collagen *α*_1_ (I) chain precursor, and keratin type II cytoskeletal 1 too.

## 4. Conclusion

Peptide mapping after specific enzymatic cleavage followed by HPLC/MS/MS method was found to be a reliable method for assaying the saiga horn proteins. There was developed an HPLC-MS/MS method for identification of saiga horn protein. This method allows to analyse proteins in individual samples of saiga horn. It can be concluded that basic proteins are keratins and collagen. The proteins presented in all samples are keratin type I microfibrillar (from *Ovis aries*, sheep), keratin type II microfibrillar (from *Ovis aries*), collagen type I (*α*_1_) from *Bos taurus*, bovine), and collagen type I (*α*_2_) (from *Bos taurus*). Free amino acids we determined in all samples are nontreated by enzyme.

## Figures and Tables

**Table 1:** Samples of saiga horn.

Age (years)	Section of horn		
	Base part	2/3	Tip
**1.7**			1.7 PGV
			1.7 DGV
**2.5**	DGN 2.5	DGV II^2^	
**3**	3 DGN		3 DGV

**Table 2:** List of identified saiga proteins. Sample 1.7 PGV, 1.7 DGV, 3 DGN, 3 DGV, 2.5 DGN, DGV II^2^ digested by trypsin.

Sample	Protein name	Distinct peptides
1.7 PGV	Keratin, type I microfibrillar 48 kDa, component 8C1—*Ovis* aries (sheep)	19
	Keratin, type II microfibrillar, component 5—*Ovis* aries (sheep)	12
	Collagen *α*_1_ (I) chain precursor—Bos taurus (bovine)	10
	Collagen *α*_2_ (I) chain precursor—Bos taurus (bovine)	11
	Keratin, high-sulfur matrix protein, IIIB—*Ovis* aries (sheep)	3
	Keratin, type II cytoskeletal 1—homosapiens (human)	4
	Zinc finger protein 643—homosapiens (human)	1

1.7 DGV	Keratin, type I microfibrillar 48 kDa, component 8C1—*Ovis* aries (sheep)	17
	Keratin, type II microfibrillar, component 5—*Ovis* aries (sheep)	9
	Keratin, type II cytoskeletal 1—Homosapiens (human)	3
	Collagen *α*_1_ (I) chain precursor—Bos taurus (bovine)	2
	Collagen *α*_2_ (I) chain precursor—Canis familiaris (dog)	2

3 DGN	Keratin, type I microfibrillar 48 kDa, component 8C1—*Ovis* aries (sheep)	19
	Keratin, type II microfibrillar, component 5—*Ovis* aries (sheep)	16
	Keratin, high-sulfur matrix protein, IIIB—*Ovis* aries (sheep)	4
	Collagen *α*_1_ (I) chain precursor—Bos taurus (bovine)	5
	Keratin, type II cytoskeletal 5—Bos taurus (bovine)	2

3 DGV	Keratin, type I microfibrillar 48 kDa, component 8C1—*Ovis* aries (sheep)	17
	Keratin, type II cuticular Hb3—homosapiens (human)	11
	Keratin, type II cytoskeletal 1—homosapiens (human)	1

2.5 DGN	Keratin, type I microfibrillar 48 kDa, component 8C1—*Ovis* aries (sheep)	11
	Keratin, type II microfibrillar, component 5—*Ovis* aries (sheep)	7
	Keratin, type II cytoskeletal 1—homosapiens (human)	2
	Keratin, type I cytoskeletal 10—homosapiens (human)	2

DGV II^2^	Keratin, type II cytoskeletal 1—homosapiens (human)	9
	Keratin, type I microfibrillar 48 kDa, component 8C1—*Ovis* aries (sheep)	9
	Keratin, type II microfibrillar, component 7C—*Ovis* aries (sheep)	7
	Keratin, type I cytoskeletal 10—homosapiens (human)	6
	Keratin, type I cytoskeletal 9—homosapiens (human)	6
	Collagen *α*_1_ (I) chain precursor—Bos taurus (bovine)	4
	Collagen *α*_2_ (I) chain precursor—canis familiaris (dog)	3
